# Optimal timing for the resection of pulmonary metastases in patients with colorectal cancer

**DOI:** 10.1097/MD.0000000000019144

**Published:** 2020-02-28

**Authors:** Kazunosuke Yamada, Daigo Ozawa, Ryouichi Onozato, Masaki Suzuki, Atsushi Fujita, Hitoshi Ojima

**Affiliations:** a Department of Gastroenterological Surgery; b Department of general thoracic surgery, Gunma Prefectural Cancer Center, Oota-shi, Gunma, Japan.

**Keywords:** colorectal neoplasms, metastasectomy, pulmonary metastasis

## Abstract

This study aims to clarify the surgical treatment time of pulmonary metastasis in patients with colorectal cancer.

Early relapse after resection of pulmonary metastasis is often encountered when the interval from the detection of pulmonary metastasis to pulmonary metastasectomy was short.

In this retrospective analysis, data of patients with colorectal cancer who underwent surgical treatment of pulmonary metastasis at the Gunma Prefectural Cancer Center, Gunma, from April 2001 through September 2018 were evaluated. The patients were divided into 2 groups. We examined the interval period from the diagnosis of pulmonary metastasis to pulmonary metastasectomy. This period was divided into every 3 months, and the prognosis of each group was compared with clarify the appropriate timing of pulmonary metastasectomy.

The primary endpoints were 5-year overall survival and recurrence-free survival rates.

The most significant difference was observed when the cutoff value was 9 months (5-year recurrence-free survival 45.8% vs 85.6%, *P* < .01). No significant difference was found in any background factors between the 2 groups. Twenty-five patients (34.7%) experienced recurrence after pulmonary metastasectomy. The most common site of recurrence was the lung (48%). Among the 12 cases of recurrence of pulmonary metastasis, 11 cases belonged to the <9 months group. A multivariable survival analysis found that the interval period of <9 months was a significant predictor of recurrence.

Our study suggests that clinical follow-up for 9 months prior pulmonary metastasectomy in colorectal patients would improve the prognosis.

## 1 Introduction

Approximately 12% of patients with colorectal cancer (CRC) are diagnosed with stage IV cancer.^[1]^ With recent advances in systemic chemotherapy and improved survival of patients with stage IV CRC,^[2,3]^ a more aggressive pulmonary resection has become a standard strategy to address pulmonary metastasis when R0 resection, or curative resection, can be achieved.^[4,5]^ In several series, 5-year survival rates ranging from 37.1% to 70.0% had been reported.^[6–10]^

In some patients with CRC where lungs are the only site of metastasis, although randomized studies are lacking, many retrospective studies suggested improved survival with metastasectomy and curative treatment of the primary CRC. However, early relapse after resection of pulmonary metastasis has often been encountered when the interval from the detection of pulmonary metastasis until pulmonary metastasectomy was short.^[11]^ Given the limited information on this theme, the present study aimed to clarify the timing of surgical treatment of pulmonary metastasis in patients with CRC.

## 2 Materials and methods

### 2.1 Design

This study has a retrospective cohort design.

### 2.2 Patients and methods

In this study, data of patients with CRC who underwent surgical treatment of pulmonary metastasis at the Gunma Prefectural Cancer Center, Gunma, from April 2001 through September 2018, were retrospectively analyzed. Informed consent was obtained from patients included in this study. We excluded patients with multiple primary cancers. After colorectal surgery, patients were regularly evaluated by chest computed tomography (CT) and abdomen CT every 6 months. Serum carcinoembryonic antigen (CEA) level was routinely checked at every 3 months. When recurrence was suspected, we tried to obtain histological or unequivocal radiological proof.

If pulmonary recurrence was considered resectable, aggressive surgical resection was carried out. The indication of pulmonary metastasectomy were control of the primary CRC, no extrapulmonary metastases, completely resectable lung metastases at preoperative imaging studies, and sufficient cardiopulmonary reserve for pulmonary metastasectomy.^[12]^ After pulmonary metastectomy, follow-up was performed in the same way as after colorectal surgery. The medical records of all the remaining patients were then reviewed for demographic information; tumor location; staging of primary cancer, tumor budding, pre-thoracotomy CEA level; disease-free interval (DFI) before development of pulmonary metastases; treatment of extrathoracic recurrences; extent, size, and treatment of thoracic metastases; intrathoracic lymph node (LN) metastases; complications; perioperative chemotherapy; recurrence-free survival (RFS); and overall survival (OS). Tumor location was categorized based on the following definition: the right colon was composed of the cecum, ascending colon, and transverse colon, and the left colon was composed of the splenic flexure, descending colon, sigmoid colon, and rectum. The tumor budding was divided in 2 groups: low budding (<5 tumor buds) per high microscopic field and high budding CRCs (≥5 buds).^[13]^ DFI was defined as the interval from the definitive treatment of the primary CRC until detection of pulmonary metastases. For patients with a history of resection of oligometastases involving another organ, the DFI was defined as the time from the resection of a previous metastasis until the first detection of the pulmonary oligometastases. These patients had to be free from other organ metastases during pulmonary metastasectomy.

Moreover, we examined the interval period from the diagnosis of pulmonary metastasis to pulmonary metastasectomy (hereinafter, referred to as interval period). This period was classified into 3 periods with 3-month interval, and prognosis in each group was compared with clarify the appropriate timing of pulmonary metastasectomy. The diagnosis date of pulmonary metastases was the date recorded by thoracic surgeons in the medical record. Metachronous presentation was defined as detection of pulmonary metastases later than 6 months following the definitive treatment of the primary CRC or a curative resection of oligometastases.

Synchronous presentation was defined as detection of the pulmonary metastases within 6 months of the definitive treatment of the primary CRC or the curative oligometastasis resection, at the same time of initial evaluation of the primary cancer or before detection of the primary cancer. For all patients, RFS was calculated from the date of pulmonary metastasectomy to the date of the first documented recurrence or death, and OS was calculated from the date of pulmonary metastasectomy to death. In cases where bilateral operations were performed, metrics were calculated from the time of the second resection.

### 2.3 Main outcome measures

The primary endpoints were 5-year OS and RFS rates.

### 2.4 Statistical analysis

SPSS for Windows (version 22.0, SPSS Inc., Chicago, IL) was used to carry out statistical analysis. Survival curves were estimated using the Kaplan–Meier method, and comparisons between curves were made by the log-rank test. Cox proportional hazard modeling was used for multivariable survival analysis. A probability level of *P* < .05 was used for statistical significance.

## 3 Results

A total of 92 patients with CRC underwent resection of pulmonary metastases. Of these, 17 patients who had multiple primary tumors and 3 patients who could not achieve disease-free status at the lung resection were excluded. Patient characteristics are summarized in Table 1. Among the 18 neoadjuvant chemotherapy cases, 7 received fluoropyrimidine, 2 received FOLFOX (5-fluorouracil/leucovorin/oxaliplatin), 5 received FOLFOX plus bevacizumab, 1 received FOLFIRI (5-fluorouracil/leucovorin/irinotecan), 2 received FOLFIRI plus bevacizumab, and 1 received cetuximab. Among the 25 adjuvant chemotherapy cases, 15 received fluoropyrimidine, 7 received FOLFOX, 1 received FOLFOX plus bevacizumab, 1 received FOLFIRI, and 1 received FOLFIRI plus bevacizumab.

**Table 1:**
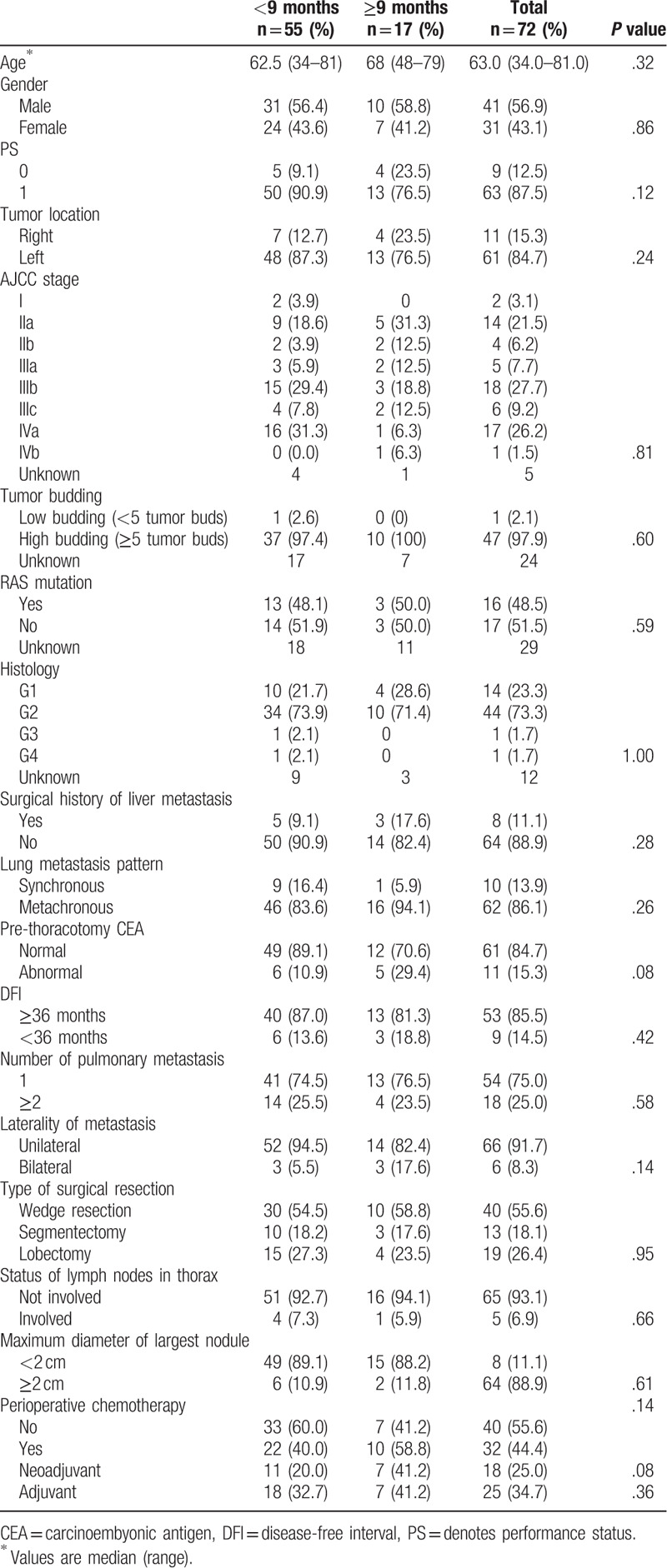
Patient characteristics.

The median follow-up of all cases was 3.9 (range, 0.5–9.9) years, and the median interval from the detection of pulmonary metastasis until pulmonary metastasectomy was 5.2 (range, 0.5–59.2) months.

**Figure 1:**
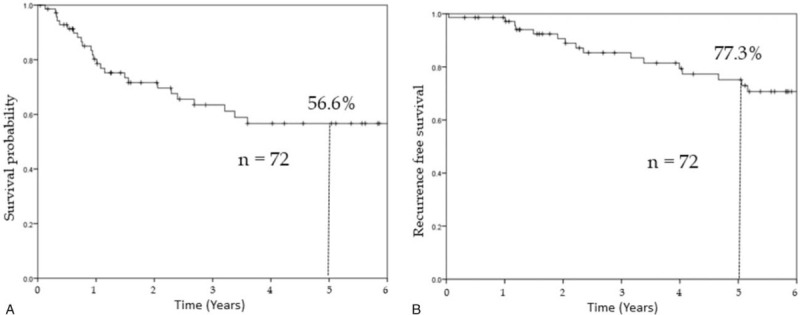
A. Kaplan–Meier overall survival curve for all patients. B. Kaplan–Meier curve for recurrence free survival for all patients.

**Figure 2:**
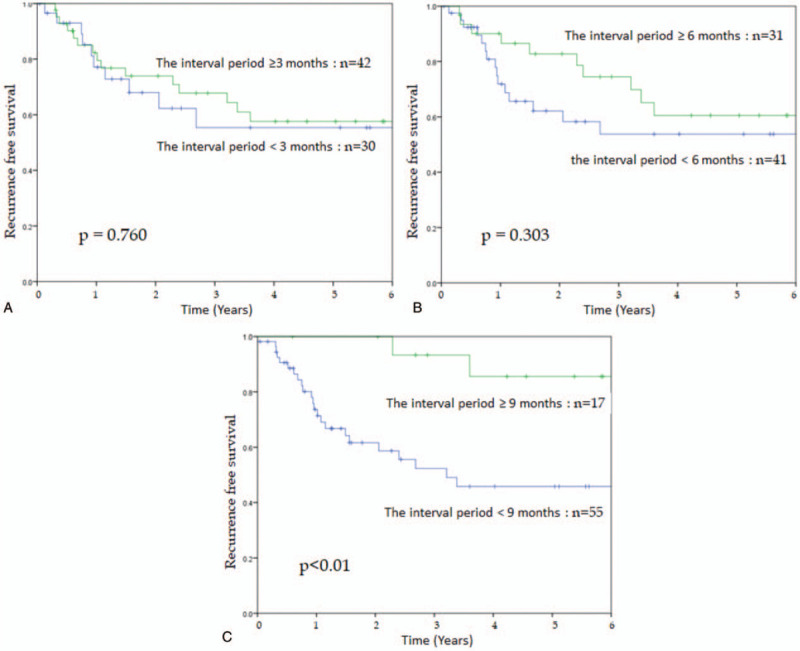
A. Kaplan–Meier curves for recurrence free survival for patients with category the interval period <3 months versus ≥3 months. B. Kaplan–Meier curves for recurrence free survival for patients with category the interval period <6 months versus ≥6 months. C. Kaplan–Meier curves for recurrence free survival for patients with category the interval period <9 months versus ≥9 months.

The 5-year OS and RFS rates were 77.3% and 56.6%, respectively (Fig. 1A and B). The cut off values of the interval period was set at 3, 6, and 9 months according to the surveillance of post curative resection in patients with stage IV CRC in Japan^[14]^ to clarify the appropriate timing of pulmonary metastasectomy, and the prognosis was examined and compared between groups (Fig. 2A–C). Since the most significant difference was observed when the cutoff value was 9 months (5-year RFS 45.8% vs 85.6%, *P* < .01), it was classified into 2 groups: <9 months group and ≥9 months group. No significant differences were found in background factors between the 2 groups (Table 1).

Twenty-five patients (34.7%) experienced recurrence after pulmonary metastasectomy. The most common site of recurrence was the lung (12 cases), followed by the liver (4 cases), multiple organs (3 cases), distant LN (2 cases), ovary, hilar LN and breast (1 case). Among the 12 cases of recurrence of pulmonary metastases, DFI after metastectomy of 9 cases were within about 12 months, 11 cases were found in the <9 months group, and 8 cases required a second metastectomy (Table 2).

**Table 2:**
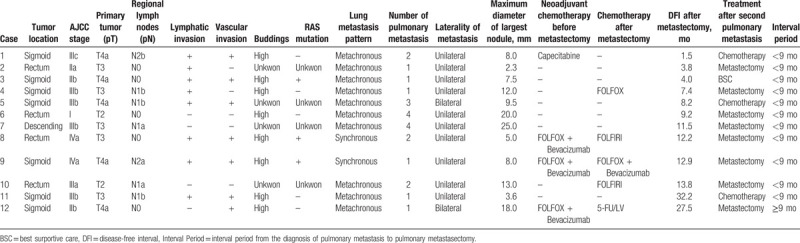
Recurrence cases of pulmonary metastasis after pulmonary metastasectomy.

In a multivariable survival analysis including these variables, the interval period of <9 months, number of pulmonary metastasis, and status of LN in the thorax were found to be significant predictors of recurrence (Table 3).

**Table 3:**
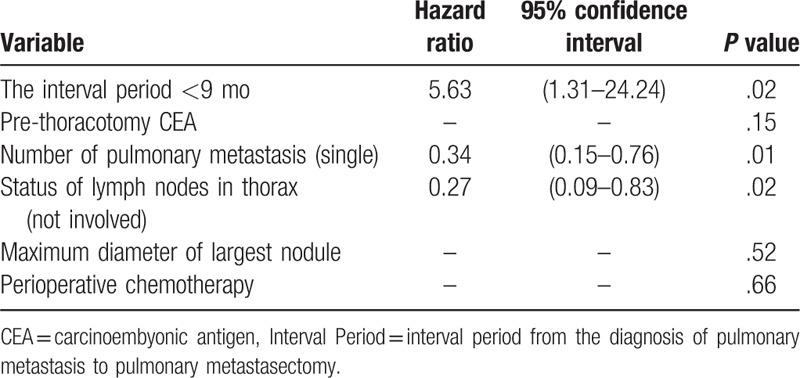
Multivariate analysis prognostic factors for disease-free survival.

## 4 Discussion

This study describes the outcome of the timing of surgical treatment of pulmonary metastasis in patients with CRC. We found that the 5-year RFS rate differed significantly depending on the interval of 9 months between the date of detection of pulmonary metastases and date in which metastasectomy was performed (5-year RFS 45.8% vs 85.6%, *P* < .01). In addition, among the 12 cases of recurrence of pulmonary metastasis, 11 cases were found in the <9 months group. Furthermore, interestingly, no significant difference was found in any background factors of the 2 groups. Our finding suggests that performing metastasectomy immediately after the diagnosis of pulmonary metastasis should be avoided.

Thus, guidelines in selecting patients who are most likely to benefit from surgical resection of pulmonary metastasectases in CRC are necessary. Moreover, this study revealed a favorable outcome of pulmonary metastasectomy in CRC. The 5-year OS rate of 77.3% and 5-year RFS rate of 56.6% in this study were better than those reported in previous multicenter studies.^[6–10]^ Some studies had shown better outcome for patients with longer DFI,^[15–19]^ solitary rather than multiple metastatic lesions,^[15,20]^ smaller size of the largest metastatic pulmonary lesion,^[20,21]^ and normal rather than elevated pre-thoracotomy CEA level.^[18,19,21,22]^ In the present study, several factors were considered to contribute to these favorable outcomes. One factor was patient selection, such as the inclusion of only patients who were free from other organ metastases during pulmonary metastasectomy, a higher proportion (75%) of patients who had a single metastasis than that reported in previous studies (45%–75%), and a smaller tumor size (median, 8 mm) relative to previous reports (median, 17–25 mm).^[6–10]^

Selection criteria had been the main focus of numerous studies evaluating factors that influence survival after metastasectomy,^[23]^ but very few reports had taken the timing of surgical treatment into account. However, many issues on the timing and appropriate indication to surgery remained opened and discussed worldwide.^[24]^ Some studies had shown better outcome for patients with longer DFI before development of pulmonary metastases.^[15–19]^ However, there can be no absolute threshold proposed along the time continuum, but indicative intervals, in which long survival cannot be anticipated, are variably suggested at 12 months,^[17]^ in the range of 19 to 39 months^[25]^ and 36 months^[23]^ in recent pooled analyses. In contrast, some studies failed to show any influence on survival based on DFI.^[26,27]^ The inconsistencies among studies may be due to the use of interoperative interval as a surrogate for the DFI in some studies, but others used the exact interval from the primary colorectal surgery to the time when the metastatic lesions were first detected after the primary surgery.^[23]^

Only 2 reports have directly analyzed the outcome based on the diagnostic interval between diagnosis and pulmonary metastasectomy.^[11,28]^ They recommend an interval of at least 3 months between the detection of pulmonary metastases and metastasectomy, in a retrospective analysis of 68 patients which shows a significantly better survival for patients with delayed operation. However, these papers focused on numerous primary cancers besides CRC, such as kidney cancer, head and neck cancer, lung cancer, tongue cancer, uterine cancer, liver cancer, etc; thus, it is doubtful whether their results can be applied to the clinical presentation of CRC. To our knowledge, the present study is the first to report on the timing of surgical treatment of pulmonary metastasis in patients with CRC.

Some surgeons have recommended performing surgery as soon as the patient is fit for surgery. This approach is often influenced by the fear that metastases could generate new metastases or the concern for a rapid local tumor progression. Kruger et al^[29]^ summarized several studies about metastasis in metastases in a review article. They emphasized that controversial but circulating tumor cells are traceable months and even years after complete resection of the primary tumor; moreover, disseminated tumor cells can recirculate from the bone marrow^[30,31]^ and the only experimental proof that metastases themselves metastasize has lost its convincing character^[32]^ from the present-day perspective. Furthermore, the authors concluded that it appears justified to perform a delayed operation, if the indication for resection is questionable due to a high risk of early multilocal relapse.^[29]^ These results may suggest that scheduling the operation immediately after diagnosis of pulmonary metastasis should be avoided. In other words, early operation is a good indication in case of operable pulmonary metastasis cases, when the number of metastases is low compared with that identified by imaging examinations >9 months ago.

The finding that we could not obtain the benefit of chemotherapy prior or after metastasectomy has to be interpreted with caution. Evidence supporting perioperative chemotherapy of pulmonary metastasis in patients with CRC is limited. No randomized controlled trials have evaluated this patient population. In a review article, Guerrera et al^[33]^ summarized 6 retrospective cohort studies about perioperative chemotherapy of resectable lung metastases in patients with CRC. They concluded that the current evidence does not support the administration of unselective perioperative chemotherapy in such patients. Moreover, JSCCR guidelines do not mention the treatment policy because the efficacy and safety of perioperative chemotherapy for distant metastatic lesion in cases of CRC have not yet been established.^[14]^ In contrast, the National Comprehensive Cancer Network recommends that active systemic therapy regimen for metastatic disease can be given before, between, or after resections.^[34]^

We acknowledge that this study has several limitations that affect our conclusions. Our treatment policy of pulmonary metastasis in patients with CRC confirms no potential metastatic disease or recurrence by chemotherapy first and then considers the possibility of surgery. However, this was not the case if the patient was referred for thoracic surgery in our institution directly from other institution with a request for early pulmonary resection. Therefore, only 18 patients (25.0%) underwent neoadjuvant chemotherapy. Furthermore, the information on the subsequent therapy was unclear as the referral was managed at the original facility after surgery. These reasons could influence our conclusions. Given the lack of robust data to support its use, perioperative chemotherapy for resected lung metastases in CRC should be evaluated in the context of a randomized controlled trial.

### 4.1 Limitations

This study had several limitations, including a small sample size and selection bias. It was difficult to demonstrate directly whether the interval period itself contributed to the improved prognosis. Patients who underwent systemic chemotherapy without surgery were excluded from the control group, and the perioperative strategy was inconsistent. However, our results clearly showed that long-term survival could be expected in select patients with CRC with an interval period ≥9 months. In the light of our results supporting the role of the interval period, randomized controlled trials are required to assess the practical interval period taking chemotherapy into account for these patients.

## 5 Conclusion

Pulmonary metastasectomy for CRC is an effective treatment and should be considered because it can provide an opportunity for cure in selected patients. Our study suggests that clinical follow-up for 9 months prior pulmonary metastasectomy in colorectal patients would improve the prognosis. Further prospective study is required for investigating predictive factor for pulmonary metastasis in patients with CRC and should take into consideration the interval period.

## Acknowledgments

The authors would like to acknowledge Dr. Imai, department of respiratory medicine, Gunma cancer center, for his help in interpreting the significance of the results of this study, and Enago (www.enago.jp) for the English language review.

## Author contributions

**Conceptualization:** Kazunosuke Yamada, Ryouichi Onozato.

**Data acquisition**: Masaki Suzuki, Ryouichi Onozato, Atsushi Fujita.

**Data analysis/interpretation**: Daigo Ozawa.

**Data curation:** Masaki Suzuki, Atsushi Fujita.

**Drafting of manuscript**: Kazunosuke Yamada.

**Formal analysis:** Daigo Ozawa.

**Manuscript preparation final approval**: Hitoshi Ojima.

**Research conception/design**: Kazunosuke Yamada.

**Writing – original draft:** Kazunosuke Yamada.

**Writing – review & editing:** Hitoshi Ojima.

Kazunosuke Yamada orcid: 0000-0001-7289-0880.

Daigo Ozawa orcid: 0000-0002-4216-6477.

Ryouichi Onozato orcid: 0000-0002-5509-0942.
